# The Unique Blood Pressures and Pulsatility of LVAD Patients: Current Challenges and Future Opportunities

**DOI:** 10.1007/s11906-017-0782-6

**Published:** 2017-10-18

**Authors:** Francesco Castagna, Eric J. Stöhr, Alberto Pinsino, John R. Cockcroft, Joshua Willey, A. Reshad Garan, Veli K. Topkara, Paolo C. Colombo, Melana Yuzefpolskaya, Barry J. McDonnell

**Affiliations:** 1 Division of Cardiology, Columbia University Medical Center, New York City, NY USA; 2 Department of Medicine, Yale New Haven Health, Bridgeport, CT USA; 3 School of Health Sciences, Cardiff Metropolitan University, Cardiff, CF5 2YB UK; 4 Department of Neurology, Columbia University Medical Center, New York City, NY USA

**Keywords:** Left ventricular assist device, Blood pressure, Continuous flow, Mechanical circulatory support, Hemodynamics, Heart failure

## Abstract

An increasing number of end-stage heart failure patients are now implanted with continuous-flow left ventricular assist devices (CF-LVADs). Although this therapeutic approach is associated with improved clinical outcomes, continuous flow physiology reduces arterial pulse pressure and pulsatility to an extent that is unique to this population. Recent data suggest that high blood pressure (BP) contributes to life-threatening complications such as pump thrombosis and stroke of CF-LVAD patients. However, limited understanding of the distinct hemodynamics of these pumps makes measurement and, consequently, medical management of BP quite challenging. Here, we review the evolution of LVAD design, the impact of CF-LVAD flow, and “artificial pulse” technology on hemodynamics and BP measurement, as well as suggest new approaches for the assessment and interpretation of the unique physiology of modern LVADs.

## Introduction

More than five million Americans are currently suffering from heart failure (HF) [1], a chronic and progressive condition characterized by the reduced ability of the heart to generate adequate cardiac output for tissue perfusion. The prevalence of this disease is rapidly increasing due to the combination of an aging population and improvements of treatments that prolong patients’ life expectancy with this disease [2]. Over the past three decades, the pharmacological management of HF has undergone tremendous progress, thus substantially improving the outcomes associated with the HF diagnosis, with the currently recommended regimen capable of reducing HF-related mortality by an astonishing 63% [3]. However, a small proportion of patients, estimated 150,000–250,000 people in the USA, will progress despite optimal medical therapy and require advanced treatments with cardiac replacement therapy [4]. Heart transplantation is the gold standard option for these patients [5], providing superior survival (median survival of approximately 11 years [6]), but with the chronic limitation of available donor hearts (only ~ 2800 patients underwent a heart transplantation over the last year in the USA). Thus, the development of mechanical circulatory support (MCS) and left ventricular assist device (LVAD) technology has come as a timely alternative to fill that gap. These devices are mechanical pumps that vicariate the failing myocardium by creating artificial blood flow from the left ventricle (LV) that generates the cardiac output required for adequate tissue perfusion. At present, a significant number of HF patients waiting for a suitable donor heart (or ineligible for a transplant) are implanted with a LVAD as a temporary substitute or definitive alternative to heart transplantation [4].

Over the past decade, close to 20,000 patients have been supported by LVADs either as a temporary substitute or definitive alternative to heart transplantation, with comparable short-term survival [4]. However, prior to the LVAD technology being considered a true alternative to heart transplantation, several obstacles need to be addressed including the adverse event profile that remains palpable. Recent data suggest that blood pressure (BP) management is at the crux of life-threatening complications such as pump thrombosis and stroke [7]. Thus, measurement, treatment, and choice of BP targets are of critical importance in the clinical management of patients on LVAD support.

## Advances in LVAD Technology

The field of MCS has rapidly evolved over the past two decades. First-generation LVADs (HeartMate XVE, Thoratec Corp., Pleasanton, CA, USA) were pulsatile devices that attempted to mimic the native pumping of the normal heart, providing enhanced arterial pulsatility. However, limited durability and reliability of the pump led to marginal improvements in patients’ survival. Contemporary second-generation LVADs are all continuous flow (CF-LVADs), and although they differ in the way they move blood within the pump (axial vs. centrifugal), mechanistically, they are quite similar. A CF-LVAD consists of the inflow cannula that sits in the left ventricular (LV) cavity, the pump impeller that rotates at a constant pre-set speed, and the outflow graft that continuously delivers blood into the aorta, augmenting the circulation throughout the entire cardiac cycle with low to no arterial pulsatility. Although longevity of the pump and patients’ survival have substantially improved in CF-LVADs compared to HeartMate XVE [8], they are still ridden with a significant adverse event profile, which can be, at least in part, attributed to the diminished pulsatility that these pumps provide. Prior reports suggest that reduced pulsatility may contribute to ischemic and hemorrhagic stroke [9], vascular dysfunction, increased matrix metalloproteinase expression [10] and oxidative stress [11], the development of arteriovenous malformations-especially in the gastrointestinal tract [12], as well as increased aortic stiffness [13]. Most recently, a new, third-generation centrifugal flow LVAD (HeartMate 3, Abbott Inc., Abbott Park, IL, USA), has reintroduced the feature of pulsatility (artificial pulse), with the main goal to reduce the rate of pump thrombosis [14]. The recently published short-term cohort results of the Multicenter Study of MagLev Technology in Patients Undergoing Mechanical Circulatory Support Therapy with HeartMate 3 (MOMENTUM 3) trial demonstrated the absence of pump thrombosis and a trend toward the reduction of non-disabling stroke rate in HeartMate 3 recipients [15]. The HeartMate 3 is currently approved in the EU (CE mark in 2015) and FDA approved as BTT and as bridge to recovery in the USA (August 2017) [16].

## Axial vs. Centrifugal CF-LVADs

Currently, FDA-approved CF-LVADs are the HeartMate II (Abbot Inc., Abbott Park, IL, USA), as bridge to transplant (BTT) and destination therapy (DT), and the HVAD (Medtronic, Inc., Minneapolis, MN, USA) for BTT indication only. The primary difference between the two devices is in the impeller design as it relates to the angle between the impeller outflow and its axis of rotation [17]. HeartMate II is an axial-flow CF-LVAD, with the impeller axis that is parallel to the outflow, while HVAD is a centrifugal-flow device where the two axes are perpendicular. This difference is reflected in the relationship between head pressure gradient (the pressure difference between the inflow cannula and the outflow graft pressures) and pump flow. While both types of CF-LVADs are sensitive to changes in head pressure, centrifugal-flow pumps are more susceptible to such changes when compared to axial flow [18]; therefore, rapid changes in afterload (i.e., blood pressure) could lead to a more substantial reduction of blood flow in centrifugal pumps.

## Blood Pressure and Clinical Outcomes in LVAD Patients

Accurate BP measurement and control has a critical importance in identification and management of CF-LVAD patients. Elevated BP has been associated with CF-LVAD-related complications, including stroke and pump thrombosis [19, 20]. Suggested BP targets are much lower than what has been shown to convey risk for cerebrovascular events in the general population, reflecting the peculiar hemodynamic and pathophysiologic consequences of CF-LVAD’s design. Current International Society of Heart and Lung Transplantation guidelines [21] recommend to maintain MAP below 80 mmHg and to use HF drugs for BP management (Level of Evidence C).

Several mechanisms may account for the association between hypertension and adverse events in CF-LVADs [22]. Elevated BP reduces LVAD flow by an increase in afterload, thus promoting stasis in the pump and, eventually, thrombosis. The thrombosis itself can result in systemic thromboembolism and stroke. In the context of chronic exposure to low pulsatility and therefore reduced endothelial function due to lack of optimal cyclical stress, even a small increase or surge in BP may exacerbate further endothelial fracture/damage in the cerebral microcirculation and predispose the local area to progressive vessel damage or even rupture. Of note, a high prevalence of hemorrhagic conversion, a process partly due to loss of blood-brain barrier integrity via endothelial injury and matrix metalloproteinase degeneration, has been reported in patients on CF-LVAD support compared to the general population [23]**.**

The increased afterload secondary to the elevated BP may cause reduced frequency of aortic valve opening, commissural fusion, and ultimately aortic insufficiency [22]. In both axial and centrifugal CF-LVAD patients, a high BP measured with Doppler during outpatient visits was associated with adverse events, mainly driven by progressive aortic insufficiency and hemorrhagic stroke [24]. In centrifugal CF-LVAD, MAP above 90 mmHg has been associated with stroke [25] and pump thrombosis [19]. An elevated SBP at discharge after axial CF-LVAD implantation has also been linked to an increased risk of stroke [20].

Though BP is associated with stroke and pump thrombosis, it is still unclear whether high BP pre-LVAD represents a marker of hypertension to stroke while on CF-LVAD support or rather the marker of a poor BP control in the outpatient in the outpatient setting. The recent PREVENtion of HeartMateII Pump Thrombosis Through Clinical Management (PREVENT) trial protocol demonstrated a low rate of pump thrombosis (2.9% at 3 months and 4.8% at 6 months) when applying standardized implant and perioperative management techniques including MAP maintenance below 90 mmHg. However, BP levels were similar in patients with and without pump thrombosis in this study [26]. More recent data from ENDURANCE Supplemental cohort indicate that an enhanced BP management that includes home BP monitoring (target MAP < 85 mmHg) successfully reduces the incidence of stroke in patients implanted with an HVAD (centrifugal CF-LVAD) compared to the original trial results [27]. Future prospective randomized trials are therefore warranted to evaluate the effect of different BP goals and different antihypertensive regimens on clinical outcomes in CF-LVAD patients.

## The Unique Hemodynamics of CF-LVAD Patients

Accurate measurement of BP in patients implanted with CF-LVADs is traditionally considered a challenge due to the continuous flow outputs associated with these devices. Several interlinking endogenous and device-related factors determine the degree of flow and pressure oscillation observed in the individual patient. For a specified pump speed, LVAD flow varies inversely to the head pressure gradient resulting in more flow during systole and less during diastole. In end-stage HF, the reduced contractility of the failing LV translates into a diminished oscillatory flow during the cardiac cycle. As a result, systolic BP (SBP) is usually lower than that observed in the general population. However, the continuous addition of blood volume to the circulation causes a reduction in pressure decay during diastole. Thus, patients’ pulse pressure (PP [PP = SBP − DBP]) is reduced to the extent that a pulse may not be palpable via traditional means.

**Fig. 1:**
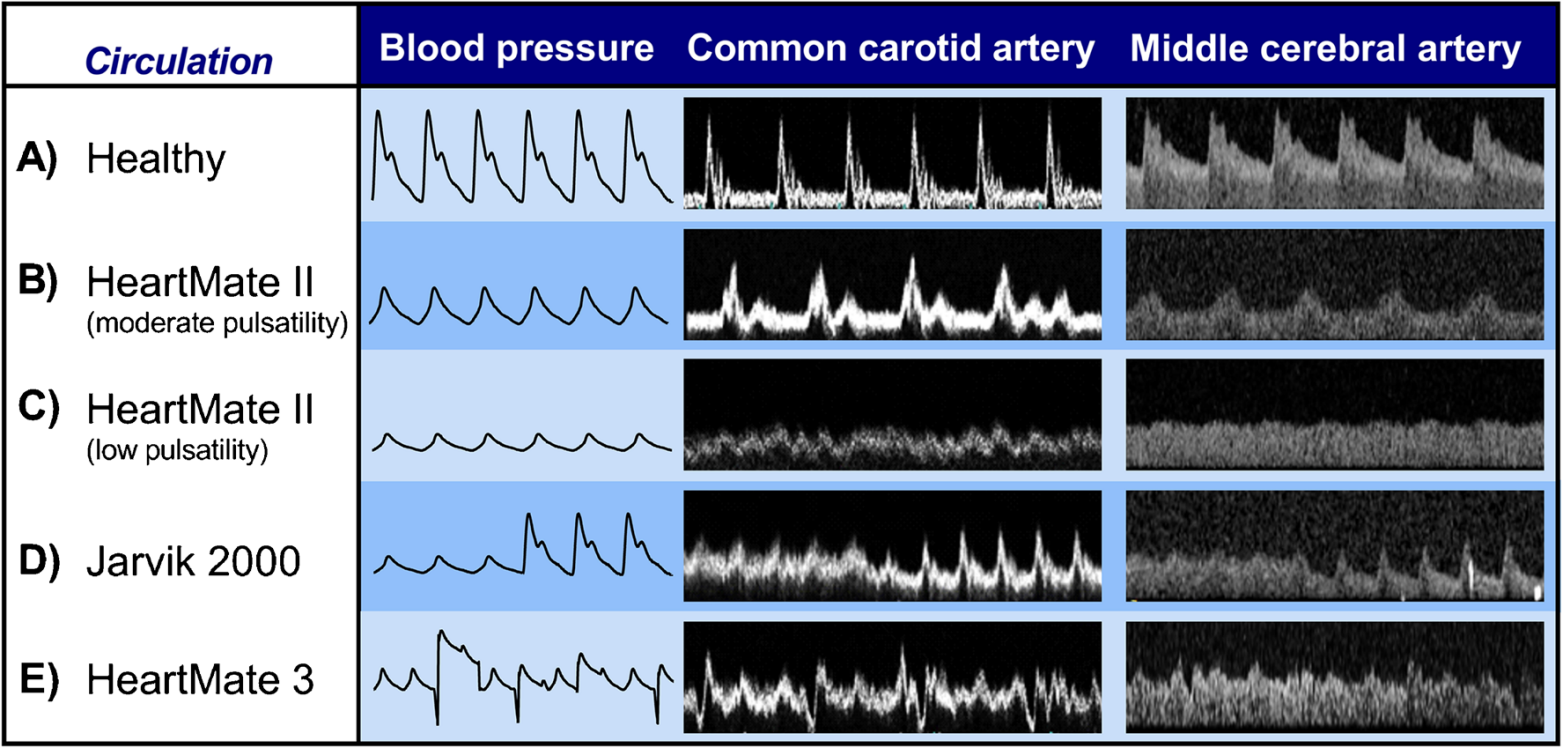
Arterial blood pressure waveforms and blood flow in the common carotid artery (CCA) and middle cerebral artery (MCA) of representative healthy and left ventricular assist device (LVAD) patients. **a** In the healthy circulation, blood pressure and CCA blood flow are pulsatile, which is somewhat reduced but still clearly present in the MCA. **b** HeartMate II with low pump speed has a moderately reduced pulse pressure and pulsatility. **c** HeartMate II with high pump speed has significantly reduced pulse pressure and pulsatility. **d** Jarvik 2000 with transition from high to low pump speed occurring every minute for a few seconds. **e** HeartMate 3 with sequential changes in pump speed occurring every 2 s (0.15 s of reduced speed by 2000 rpm below baseline and 0.20s of increased speed by 2000 rpm above baseline), also termed “artificial pulse”

Unlike the hemodynamics of healthy individuals (Fig. 1a), in CF-LVADs, the flow rate that the pump generates modulates blood flow and BP profiles. A higher LVAD speed augments aortic pressure and unloads the LV (more blood is removed by the device) reducing peak systolic pressure. Under these conditions, the aortic valve tends to remain closed and blood flow in the aorta and peripheral circulation becomes less pulsatile, since the systolic contribution of the unloaded LV is significantly reduced (Fig. 1c). The opposite is observed at lower pump speeds (Fig. 1b). The altered physiology that is associated with a change in pump speed is confirmed within the same patient when the failing heart is supported by a Jarvik 2000 device. The Jarvik 2000 is an axial CF-LVAD, which is currently undergoing a prospective, dual-armed, non-blinded (open-label), randomized clinical trial for DT indication. Every minute, it operates at a high speed for most of the time, but reduces its contribution to blood flow for few seconds to increase LV loading and promote aortic valve opening [28]. This results in a remarkable, immediate change in blood pressure and hemodynamics (Fig. 1d), emphasizing the unique physiological interplay between the CF-LVAD and the native heart.

## BP Measurement in CF-LVAD

Indwelling arterial catheters (A-line) are considered the most accurate method to measure BP in CF-LVAD patients, but its use is limited to hospitalized patients during their stay in the intensive care unit. Common automated BP monitors often fail to measure BP due to the lack of detectable flow oscillations in CF-LVAD patients. In fact, Doppler ultrasound is often used as an alternative to automated BP monitors in patients without an A-line [29]. Unfortunately, Doppler ultrasound has the major limitation of providing a single BP number, and whether this number represents the SBP or the MAP has been focus of extensive debate in the MCS scientific community [30].

To better understand why common automated BP monitors are often unable to read BP in CF-LVAD patients, it is worth to review how BP cuff systems work. A typical oscillometric BP monitor is composed of an inflatable BP cuff connected to a pressure transducer. When activated, the cuff initially inflates to a pressure above the SBP and then gradually deflates until the transducer senses an increase in oscillations (this point corresponds to SBP); the system continues to deflate until the transducer senses the maximum oscillation amplitude (this point corresponds to MAP). Using SBP and the MAP values, the system can estimate DBP. In CF-LVAD patients, the narrow PP critically reduces the difference between the first increase in oscillation (i.e., SBP) and the point of maximum oscillation (i.e., MAP). Background noise from the continuous flow pump further impairs the ability of oscillometric BP monitors to obtain a BP reading in this unique population. However, the Terumo Elemano BP Monitor (Terumo Elemano, Hatagaya, Shibuya, Japan) could overcome this limitation thanks to a slow and more sensitive cuff deflation process [30]. Unfortunately, the production of this device has been discontinued since 2014. As an alternative, our group recently tested and validated the Mobil-O-Graph device (IEM, Stolberg, Germany) in measuring BP in HeartMate II patients [31]. Success rate was 82%, and a mean absolute difference for the SBP and MAP compared to A-line was 4.5 ± 0.7 and 4.0 ± 0.6 mmHg, respectively. Importantly, the Mobil-O-Graph device also allows measurement of ambulatory BP over 24 h.

## A New Generation Continuous-Flow LVADs with Artificial Pulse

**Fig. 2:**
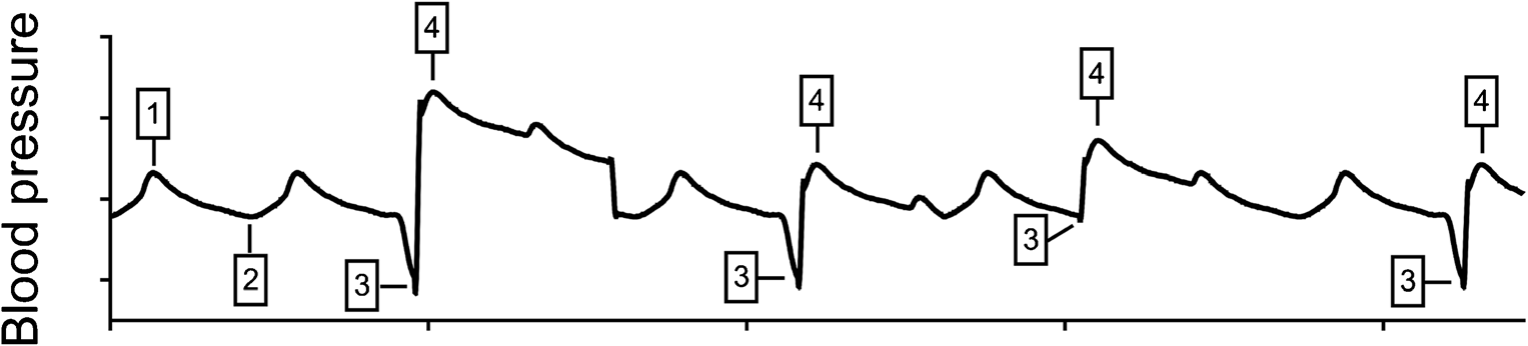
Blood pressure waveform in HeartMate 3. The arterial blood pressure waveform comprises of cardiac and LVAD-related systolic and diastolic phases: heart-systolic with the continuous flow from the LVAD (*1*), heart-diastolic with the continuous flow from the LVAD (*2*), LVAD artificial pulse-diastolic (*3*), and LVAD artificial pulse-systolic (*4*). The relationship between these components constantly varies since the “artificial pulse” is not synchronized with the native heartbeat. Repeated occurrences of *1* and *2* are not shown, but appear in intervals between the artificial pulse (*3* and *4*)

The new generation CF-LVAD with an artificial pulse (CF-LVAD^+AP^), the HeartMate 3, provides intermittent swings in flow that result in the creation of an artificial pulse. Every 2 s, this pump alternatively powers down the rotor speed by 2000 rpm for 0.15 s, then increases by 4000 rpm for 0.20 s, before returning to the set speed [32] (Fig. 1e). Importantly, these changes in speed are timed independently from the native cardiac cycle (asynchronous pulsation). As a result, BP tracings constantly vary based on the asynchronous relationship between Heart and LVAD systolic and diastolic phases. Thus, the BP profile in HeartMate 3 patients comprises of several components. The native heart and its residual contractility generate blood flow with cyclic systolic and diastolic phases (heart-systolic BP and heart-diastolic BP). The HeartMate 3 adds two additional “artificial” components: the flow reduction phase, where the device reduces the impeller speed (LVAD-diastolic BP), and the flow increase phase, where the device increases the impeller speed (LVAD-systolic BP) (Fig. 2).

Thus, measurement and interpretation of BP are more complex in HeartMate 3 due to the non-uniformity of the pressure profile. BP measurements from A-line remain the gold standard; however, appropriate non-invasive methods of measurement remain to be validated. This task might be particularly challenging in HeartMate 3 patients, since BP monitors (as well as Doppler) may not recognize and/or accurately interpret pump-specific patterns of oscillations during cuff deflation. For example, accurate calculation of MAP may require assessment over extended periods of time that include several cardiac cycles. In addition, BP monitors will not be able to recognize separately heart and LVAD systolic and diastolic BP values. This might represent an important limitation since it is conceivable that measurement of individual BP components could be relevant for the prediction of adverse events in this patient population.

## BP and Energy

In 1966, Shepard and colleagues developed a concept of energy equivalent pressure (EEP) [33], where BP is equivalent to the kinetic energy delivered from the heart to peripheral vasculature and tissues. In the general population, not on CF-LVAD support, this energy is described and simplified as SBP, DBP, or MAP due to the regularity and the repetitiveness of cardiac cycles and the relative uniformity of the BP tracing. In the case of constant flow, EEP simply approximates MAP. This concept suggests that it is appropriate to use MAP as an estimation of delivered energy in CF-LVAD patients. Of note, this is eventually what the MCS scientific community has been doing for years, using MAP values to target BP goals, in this patient population.

However, this concept may not apply in situations of erratic BP profile such as in patients implanted with a CF-LVAD^+AP^ (Fig. 1e). Here, the relationship between energy and heart/LVAD-derived BP components is more complex and does not lend itself to easy interpretations. Nevertheless, better understanding of the type of energy that CF-LVAD^+AP^ transmit to the periphery might be important in predicting and, therefore, preventing vascular damage to vital organs and thereby improve clinical outcomes.

## Conclusion

The number of CF-LVAD patients (and HeartMate 3 in particular) is expected to markedly increase over the next number of decades. It is therefore critical to meet patients’ needs by identifying valid methods to measure and manage their BP as a way to reduce morbidity and mortality. Currently, automated BP monitors (and/or Doppler) can be effectively used in the majority of patients who are implanted with an FDA-approved CF-LVADs such as the HeartMate II and HVAD. However, the HeartMate 3 offers a new challenge since it incorporates artificial pulse technology. This fixed pulse is asynchronous to the native heartbeat, and because of that, it generates an erratic BP profile that includes cardiac and LVAD-generated systolic and diastolic components. Nevertheless, accurate assessment and interpretation of the (patho)physiological consequences of this unique hemodynamic profile may play an important role in optimizing safety and efficacy of this novel CF-LVAD technology.

**Table 1:** Abbreviations and definitions

Abbreviation	Definition	Description
BP	Blood pressure	The pressure within arteries
CE mark	Conformité Européene	European Union conformity marking
CF-LVAD	Continuous-flow left ventricular assist device	Surgically implanted device to assist the failing heart, producing a continuous outflow into the main arterial circulation
CF-LVAD^+AP^	Continuous-flow left ventricular assist device with “artificial pulse”	Similar to CF-LVAD, but with a regular alteration of the continuous flow by change in pump speed
DBP	Diastolic blood pressure	The minimum blood pressure during one cardiac cycle
EEP	Energy equivalent pressure	Hemodynamic energy associated with pressure-flow waveforms
FDA	Food and Drug Administration	Federal agency responsible for drug and device approvals in the USA
LVAD	Left ventricular assist device	Mechanical device surgically implanted to support the failing heart.
MAP	Mean arterial pressure	The average blood pressure over one cardiac cycle
PP	Pulse pressure	The difference between systolic and diastolic blood pressure
SBP	Systolic blood pressure	The maximum blood pressure during one cardiac cycle

## References

[1] Go AS, Mozaffarian D, Roger VL, Benjamin EJ, Berry JD, Borden WB (2013). Heart disease and stroke statistics—2013 update: a report from the American Heart Association. Circulation.

[2] Smolina K, Wright FL, Rayner M, Goldacre MJ. Determinants of the decline in mortality from acute myocardial infarction in England between 2002 and 2010: linked national database study. BMJ (Clinical research ed). 2012;344. doi:10.1136/bmj.d8059. 10.1136/bmj.d8059 PMC3266430 22279113

[3] Burnett H, Earley A, Voors AA, Senni M, McMurray JJ, Deschaseaux C et al. Thirty years of evidence on the efficacy of drug treatments for chronic heart failure with reduced ejection fraction: a network meta-analysis. Circ Heart Fail. 2017;10(1). doi:10.1161/circheartfailure.116.003529. 10.1161/CIRCHEARTFAILURE.116.003529 PMC5265698 28087688

[4] Mancini D, Colombo PC (2015). Left ventricular assist devices: a rapidly evolving alternative to transplant. J Am Coll Cardiol.

[5] Mancini D, Lietz K (2010). Selection of cardiac transplantation candidates in 2010. Circulation.

[6] Lund LH, Edwards LB, Kucheryavaya AY, Benden C, Dipchand AI, Goldfarb S (2015). The Registry of the International Society for Heart and Lung Transplantation: thirty-second official adult heart transplantation report—2015; focus theme: early graft failure. J Heart Lung Transplant: Off Publ Int Soc Heart Transplant.

[7] Willey JZ, Boehme AK, Castagna F, Yuzefpolskaya M, Garan AR, Topkara V (2016). Hypertension and stroke in patients with left ventricular assist devices (LVADs). Curr Hypertens Rep.

[8] Slaughter MS, Rogers JG, Milano CA, Russell SD, Conte JV, Feldman D (2009). Advanced heart failure treated with continuous-flow left ventricular assist device. N Engl J Med.

[9] Starling RC, Moazami N, Silvestry SC, Ewald G, Rogers JG, Milano CA (2014). Unexpected abrupt increase in left ventricular assist device thrombosis. N Engl J Med.

[10] Gambillara V, Thacher T, Silacci P, Stergiopulos N (2008). Effects of reduced cyclic stretch on vascular smooth muscle cell function of pig carotids perfused ex vivo. Am J Hypertens.

[11] Thacher T, Gambillara V, da Silva RF, Silacci P, Stergiopulos N (2010). Reduced cyclic stretch, endothelial dysfunction, and oxidative stress: an ex vivo model. Cardiovasc Pathol: Off J Soc Cardiovasc Pathol.

[12] Demirozu ZT, Radovancevic R, Hochman LF, Gregoric ID, Letsou GV, Kar B (2011). Arteriovenous malformation and gastrointestinal bleeding in patients with the HeartMate II left ventricular assist device. J Heart Lung Transplant: Off Publ Int Soc Heart Transplant.

[13] Patel AC, Dodson RB, Cornwell WK, Hunter KS, Cleveland JC, Brieke A (2017). Dynamic changes in aortic vascular stiffness in patients bridged to transplant with continuous-flow left ventricular assist devices. JACC Heart Fail.

[14] Netuka I, Sood P, Pya Y, Zimpfer D, Krabatsch T, Garbade J (2015). Fully Magnetically levitated left ventricular assist system for treating advanced HF. J Am Coll Cardiol.

[15] Uriel N, Colombo PC, Cleveland JC, Long JW, Salerno C, Goldstein DJ (2017). Hemocompatibility-related outcomes in the MOMENTUM 3 trial at 6 months: a randomized controlled study of a fully magnetically levitated pump in advanced heart failure. Circulation.

[16] MOMENTUM 3 IDE Clinical Study Protocol.

[17] Moazami N, Fukamachi K, Kobayashi M, Smedira NG, Hoercher KJ, Massiello A (2013). Axial and centrifugal continuous-flow rotary pumps: a translation from pump mechanics to clinical practice. J Heart Lung Transplant: Off Publ Int Soc Heart Transplant.

[18] Farrar DJ, Bourque K, Dague CP, Cotter CJ, Poirier VL (2007). Design features, developmental status, and experimental results with the Heartmate III centrifugal left ventricular assist system with a magnetically levitated rotor. ASAIO J (Am Soc Artif Intern Organs: 1992)..

[19] Najjar SS, Slaughter MS, Pagani FD, Starling RC, McGee EC, Eckman P (2014). An analysis of pump thrombus events in patients in the HeartWare ADVANCE bridge to transplant and continued access protocol trial. J Heart Lung Transplant: Off Publ Int Soc Heart Transplant.

[20] Nassif ME, Tibrewala A, Raymer DS, Andruska A, Novak E, Vader JM (2015). Systolic blood pressure on discharge after left ventricular assist device insertion is associated with subsequent stroke. J Heart Lung Transplant: Off Publ Int Soc Heart Transplant.

[21] Feldman D, Pamboukian SV, Teuteberg JJ, Birks E, Lietz K, Moore SA (2013). The 2013 International Society for Heart and Lung Transplantation Guidelines for mechanical circulatory support: executive summary. J Heart Lung Transplant: Off Publ Int Soc Heart Transplant.

[22] Wasson LT, Yuzefpolskaya M, Wakabayashi M, Takayama H, Naka Y, Uriel N (2015). Hypertension: an unstudied potential risk factor for adverse outcomes during continuous flow ventricular assist device support. Heart Fail Rev.

[23] Willey JZ, Gavalas MV, Trinh PN, Yuzefpolskaya M, Reshad Garan A, Levin AP (2016). Outcomes after stroke complicating left ventricular assist device. J Heart Lung Transplant: Off Publ Int Soc Heart Transplant.

[24] Saeed O, Jermyn R, Kargoli F, Madan S, Mannem S, Gunda S (2015). Blood pressure and adverse events during continuous flow left ventricular assist device support. Circ Heart Fail.

[25] Teuteberg JJ, Slaughter MS, Rogers JG, McGee EC, Pagani FD, Gordon R (2015). The HVAD left ventricular assist device: risk factors for neurological events and risk mitigation strategies. JACC Heart Fail.

[26] Maltais S, Kilic A, Nathan S, Keebler M, Emani S, Ransom J (2017). PREVENtion of HeartMate II pump thrombosis through clinical management: the PREVENT multi-center study. J Heart Lung Transplant: Off Publ Int Soc Heart Transplant.

[27] Milano CA, Rogers JG, Tatooles AJ, Bhat G, Slaughter MS, Birks EJ, et al. The treatment of patients with advanced heart failure ineligible for cardiac transplantation with the HeartWare ventricular assist device: results of the ENDURANCE supplement trial. J Heart Lung Transplant. 36(4):S10. 10.1016/j.healun.2017.01.012.

[28] Operator Manual Jarvik 2000® Adult Ventricular Assist System, Post-Auricular Cable.

[29] Bennett MK, Roberts CA, Dordunoo D, Shah A, Russell SD (2010). Ideal methodology to assess systemic blood pressure in patients with continuous-flow left ventricular assist devices. J Heart Lung Transplant: Off Publ Int Soc Heart Transplant.

[30] Lanier GM, Orlanes K, Hayashi Y, Murphy J, Flannery M, Te-Frey R (2013). Validity and reliability of a novel slow cuff-deflation system for noninvasive blood pressure monitoring in patients with continuous-flow left ventricular assist device. Circ Heart Fail.

[31] Castagna F, McDonnell BJ, Stohr EJ, Yuzefpolskaya M, Trinh PN, Topkara VK, et al. Non-invasive measurement of peripheral, central and 24-hour blood pressure in patients with continuous-flow left ventricular assist device. J Heart Lung Transplant: Off Publ Int Soc Heart Transplant. 2017; 10.1016/j.healun.2017.02.026. 10.1016/j.healun.2017.02.026 28336236

[32] Bourque K, Cotter C, Dague C, Harjes D, Dur O, Duhamel J (2016). Design rationale and preclinical evaluation of the HeartMate 3 left ventricular assist system for hemocompatibility. ASAIO J (Am Soc Artif Intern Organs: 1992).

[33] Shepard RB, Simpson DC, Sharp JF (1966). Energy equivalent pressure. Arch Surg (Chicago, Ill: 1960).

